# Impact Of Social Network Size And Diversity On Resting EEG Patterns

**DOI:** 10.1002/alz70856_107754

**Published:** 2026-01-09

**Authors:** Acebo García‐Guerrero, Jonathan Adrian Zegarra‐Valdivia, Brenda Nadia Chino‐Vilca, Natalia Ojeda del Pozo, Ignacio Torres Aleman

**Affiliations:** ^1^ University of Deusto, Bilbao, Vizcaya, Spain; ^2^ Achucarro Basque Center for Neuroscience, Leioa, Spain; ^3^ Achucarro Basque Center for Neuroscience, Bilbao, Spain

## Abstract

**Background:**

Social interactions are integral to human well‐being, affecting cognitive functions, emotional states, and neurological processes. While prior research has assessed the impact of social networks on mental health outcomes, the neural mechanisms underlying these relationships are less understood. By analyzing the Social Network Index (SNI) alongside resting‐state electroencephalography (EEG) data, the study aims to uncover neural correlates of sociability and perceptions of loneliness.

**Method:**

A total of 26 participants (mean age = 21.93 years, SD = 4.83; 73.1% female) were included after screening for the absence of mental illness, neurological disorders, and other exclusion criteria. Participants completed Cohen's SNI and the UCLA Loneliness Scale and underwent a 5‐minute eyes‐closed resting‐state EEG recording using an ANT portable 64‐channel EEG amplifier, sampled at 2000 Hz with a band‐pass filter between 0.1 and 330 Hz. The study received ethical approval from Deusto University's ethics committee.

**Result:**

The results revealed significant correlations between sociodemographic factors, social network measures, and electrophysiological power signatures. Age was negatively associated with the SNI (older participants had contact with fewer people), and years of education were negatively linked to the network diversity component (SNInet), indicating that more educated individuals had less variety in their social circles. Specifically, the SNI correlated negatively with high beta power at temporal regions (S_highβ_T; rho = −0.639, *p* < 0.001), high beta power at temporo‐parieto‐occipital regions (S_highβ_TPO; rho = −0.674, *p* < 0.001), and beta power at temporal regions (S_β_T; rho = −0.433, *p* < 0.05). This suggests that higher high beta activity is associated with a lower volume of social relationships. When dividing participants based on the number of social networks and controlling for age, significant differences were observed in high beta power at temporo‐parieto‐occipital regions (S_highβ_TPO; F(1,22) = 12.75, *p* < 0.005, partial η^2^ = 0.367).

**Conclusion:**

The study suggests a relevant role of high beta activity in individuals' sociability and perception of loneliness. Participants with higher high beta power had fewer social relationships but reported lower feelings of loneliness. These findings indicate a complex relationship between brain activity and social engagement, warranting further research to understand the underlying neural mechanisms more specifically.

